# The Phytochemical and Biological Investigation of *Jatropha pelargoniifolia* Root Native to the Kingdom of Saudi Arabia

**DOI:** 10.3390/molecules23081892

**Published:** 2018-07-28

**Authors:** Hanan Y. Aati, Ali A. El-Gamal, Oliver Kayser, Atallah F. Ahmed

**Affiliations:** 1 Department of Pharmacognosy, Faculty of Pharmacy, King Saud University, P.O. Box 2457, Riyadh 11451, Saudi Arabia; hati@ksu.edu.sa (H.Y.A.); afahmed@ksu.edu.sa (A.F.A.); 2 Department of Pharmacognosy, College of Pharmacy, Mansoura University, El-Mansoura 35516, Egypt; 3 Technical Biochemistry, TU Dortmund University, Emil-Figge-Strasse 66, D-44227 Dortmund, Germany; oliver.kayser@tu-dortmund.de

**Keywords:** *Jatropha pelargoniifolia*, alkaloids, flavonoids, coumarinolignans, diterpenes, anti-inflammatory, analgesic, antipyretic

## Abstract

Extensive phytochemical analysis of different root fractions of *Jatropha pelargoniifolia* Courb. (Euphorbiaceae) has resulted in the isolation and identification of 22 secondary metabolites. 6-hydroxy-8-methoxycoumarin-7-*O*-β-d-glycopyranoside (**15**) and 2-hydroxymethyl *N*-methyltryptamine (**18**) were isolated and identified as new compounds along with the known diterpenoid (**1**, **3**, **4**, and **7**), triterpenoid (**2** and **6**), flavonoid (**5**, **11**, **13**, **14**, and **16**), coumarinolignan (**8**–**10**), coumarin (**15**), pyrimidine (**12**), indole (**17**, **18**), and tyramine-derived molecules (**19**–**22**). The anti-inflammatory, analgesic, and antipyretic activities were evaluated for fifteen of the adequately available isolated compounds (**1**–**6**, **8**–**11**, **13**, **14**, **16**, **21**, and **22**). Seven (**4**, **6**, **10**, **5, 13**, **16**, and **22)** of the tested compounds showed a significant analgesic effect ranging from 40% to 80% at 10 mg/kg in two in vivo models. Compound **1** could also prove its analgesic property (67.21%) when it was evaluated on a third in vivo model at the same dose. The in vitro anti-inflammatory activity was also recorded where all compounds showed the ability to scavenge nitric oxide (NO) radical in a dose-dependent manner. However, eight compounds (**1**, **4**, **5**, **6**, **10**, **13**, **16**, and **22**) out of the fifteen tested compounds exhibited considerable in vivo anti-inflammatory activity which reached 64.91% for compound **10** at a dose of 10 mg/kg. Moreover, the tested compounds exhibited an antipyretic effect in a yeast-induced hyperthermia in mice. The activity was found to be highly pronounced with compounds **1**, **5**, **6**, **10**, **13**, and **16** which decreased the rectal temperature to about 37 °C after 2 h of the induced hyperthermia (~39 °C) at a dose of 10 mg/kg. This study could provide scientific evidence for the traditional use of *J. pelargoniifolia* as an anti-inflammatory, analgesic, and antipyretic.

## 1. Introduction

Euphorbiaceae is considered as one of the largest families of flowering plants which includes approximately 7800 species that are distributed among 300 genera and five subfamilies in tropical and subtropical regions [1,2]. Among the main genera of this family, *Jatropha* L. is represented by approximately 200 species [2]. *Jatropha* species are used in folk medicine to treat various diseases, such as skin inflammation, eye infection, chest pain, stomach pain, itching, and as a vermifuge, or as ornamental plants and energy crops in Latin America, Africa, and Asia [3]. *J. gossypiifolia*, *J. elliptica*, *J. curcas*, and *J. mollissima*, among other species of *Jatropha*, have been reported for their chemical constituents, biological activities, and medicinal uses [4]. *Jatropha glauca*, *J. curcas*, *J. spinose*, and *J. pelargoniifolia* are the only four species that are distributed in Saudi Arabia and are employed as traditional herbal medicines, owing to their anti-inflammatory, antioxidant, antiseptic, and analgesic properties [5,6]. 

*J. pelargoniifolia* Courb. of the current study is grown as a shrub and is widely known as “Obab” in Arabic. It is widely distributed in East Tropical Africa (Sudan, Eritrea, Ethiopia, Somalia, and Kenya) and the Arabian Peninsula (Yemen, Oman, and Saudi Arabia) [7]. The plant is sometimes collected from the wild for local medicinal use, especially the petiole sap which is applied to treat ulcers, severe skin inflammation, and for wound healing [7].

Previous phytochemical studies on the plants belonging to the genus *Jatropha* revealed a broad range of isolated secondary metabolites, such as diterpenoids, triterpenoids, non-conventional coumarino-lignans, alkaloids, coumarins, flavonoids, cyclic peptides, and steroids [8,9]. However, accordingly reviewed by Zhang et al. [8], the main compounds isolated from *Jatropha* genus are the terpenoids. *J. gossypiifolia* was subjected to extensive phytochemical studies that resulted in the isolation of many secondary metabolites, such as propacin, venkatasin, citlalitrione, ricinine, apigenin, jatropholones A& B, and jatrophone [4]. Moreover, curcusones A–D, taraxerol, nobiletin, curacyclines A & B, as well uracil, have been isolated from *J. curcas*. [5,6,8]. Additionally, many reported studies showed the isolation of multidione, multifidone, multifolone, and multifidol glucoside from *J. multifida,* while from *J. podagrica, there* was japodic acid, erythrinasinate, γ-sitosterol, japodagrin, and podacyclines A & B [3,8,9]. This is indeed a reflection of the versatility of the enzymatic system that is present in Euphorbiaceous plants, however nothing was reported regarding *J. pelargoniifolia*. Thus, it was of interest to explore the active constituents and their biological activity to provide evidence for the traditional use of *J. pelargoniifolia.*

## 2. Result and Discussion

### 2.1. Isolation of Compounds

**Figure 1:**
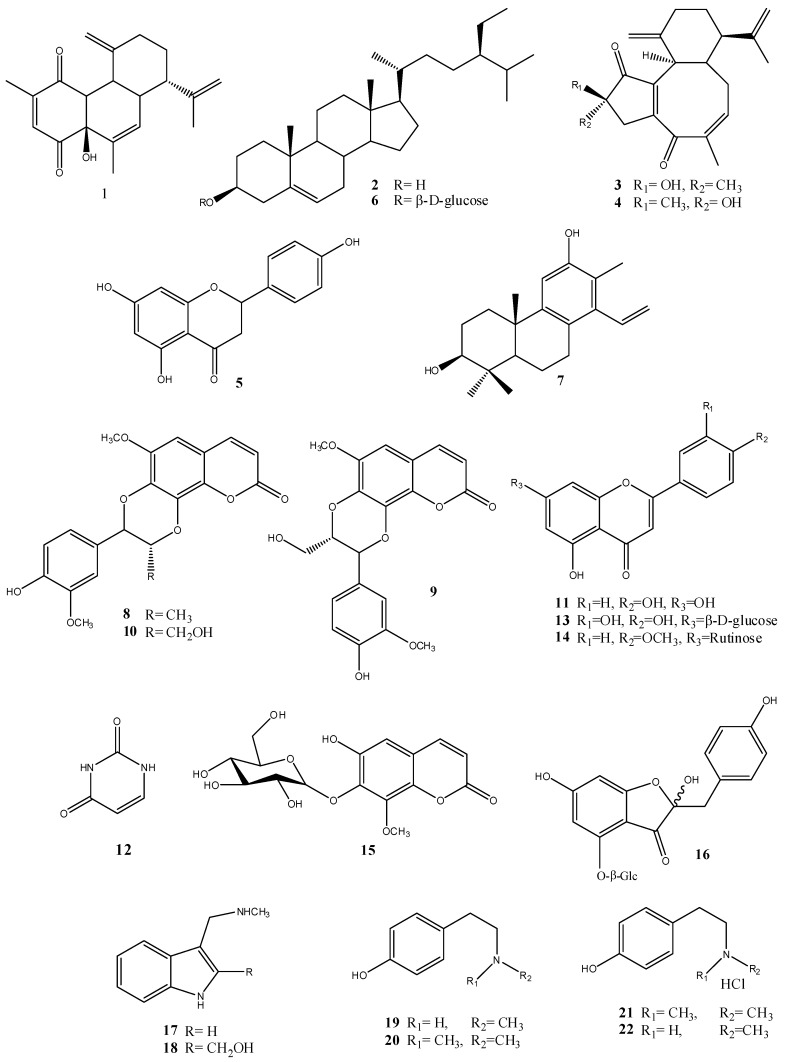
Chemical structures of the compounds isolated from the roots of *Jatropha pelargoniifolia*.

The alcoholic extract of *J. pelargoniifolia* roots powder was successively partitioned with petroleum ether (60 °C), dichloromethane (DCM), ethyl acetate (EtOAc), and then *n*-butanol (*n*-BuOH) to give the correspondent organic fractions. Each fraction was subjected to chromatographic separation on normal and reversed phase (RP) silica gel to yield compounds **1**, **6**, **7**, **10**, **11**, and **16** from petroleum ether, DCM_2_, and EtOAc fractions, respectively. Furthermore, the organic extract that was obtained after an acid-base treatment of the roots powder was isolated on a normal silica gel column which was followed by purification on RP-HPLC and/or crystallization to afford compounds **17**–**22** (Figure 1)*.*

### 2.2. Structure Elucidation

**Figure 2:**
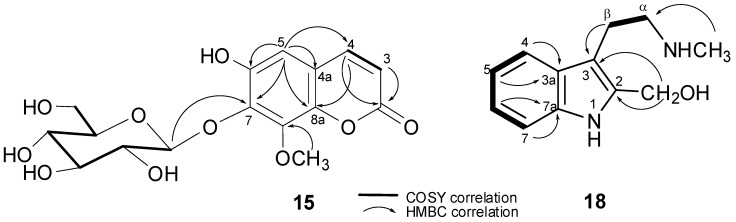
Selected heteronuclear multiple bond correlation (HMBC) and correlation spectroscopy (COSY) correlations of compounds **15** and **18**.

The new compound **15** was obtained as white crystals. The NMR and ESIMS (Electronspray Ionization Mass Spectrometry) data established the molecular formula of **15** to be C_16_H_18_O_10_. The IR absorption bands at max 3349, 1719, and 1625 cm^−1^ suggested the presence of hydroxyl, ester carbonyl, and aromatic functionalities, respectively. Furthermore, the ^13^C nuclear magnetic resonance (NMR) spectrum of **15**, which was measured in deuterated methanol (CD_3_OD, displayed sixteen signals of nine sp^2^ and seven sp^3^ carbons (including that of a methoxyl group). Its ^1^H NMR spectrum showed a pair of ortho-coupled protons at δ_H_ 6.26 and 7.88 (each, 1H, d, *J* = 9.5 Hz) as was observed by ^1^H-^1^H correlated spectroscopy (COSY) that was assignable to H-3 and H-4 of an α-pyrone ring system of a coumarin, respectively [10]. This was further evidenced from the ^13^C NMR carbon signals of the -pyrone at δ_C_ 163.5 (C, C-2), 146.5 (CH, C-4), 144.4 (C, C-8a), 116.2 (CH, C-3), and 112.7 (C, C-4a) (Table 1). Moreover, the single aromatic singlet appearing at δ_H_ 7.00 (1H, s) suggested **15** to be a trisubstituted coumarin. Six proton signals at δ_H_ 3.30–4.99 ppm, a doublet of an anomeric proton at δ_H_ 4.99 (1H, d, *J* = 7.8 Hz), and six ^13^C NMR signals at δ_C_ 106.2 (CH, C-1′), 75.5 (CH, C-2′), 77.8 (CH, C-3′), 71.0 (CH, C-4′), 78.5 (CH, C-5′), and 62.2 (CH_2_, C-6′) indicated the presence of a β-D-glucopyranosyl substituent. An aromatic methoxy substituent (δ_H_/δ_C_ 3.91/57.0) was also revealed. The ^3^*J*_CH_ correlations that were observed in the heteronuclear multiple bond correlation (HMBC) spectrum linked these two substituents to the coumarin carbons at δ_C_ 133.2 and 147.5, respectively (Figure 2). Thus, a hydroxy group should represent the third substituent on the coumarin carbon at δ_C_ 145.7. The long-range correlations (HMBC) that were found from H-5 (δ_H_ 7.00, 1H, s) to the carbons at δ_C_ 146.5 (C-4), 145.7, C-8a (δ_C_ 144.4), and δ_C_ 133.2 (C-7) indicated that C-8 (δ_C_ 147.5) is the position of the methoxyl group. To confirm the locations of the glucosyl and hydroxyl groups, the NMR data of **15** were further compared to those of 5-hydroxy-7-methoxycoumarin-8-*O*-β-d-glucoside and other closely related coumarin derivatives that were previously isolated from *Daphne pseudo-mezereum* [11] and *Tetraphis pellucida* [12], respectively. The structure of compound **15** was thus established as a new natural product and was identified as 6-hydroxy-8-methoxy coumarin-7-*O*-β-d-glycopyranoside. 

Compound **18** was isolated from the organic extract of the acid-base treated root powder as white needle-shaped crystals. It produced a positive Dragendorff’s test, indicating its alkaloid nature. The IR absorption band with a spike at max 3309 cm^−1^ suggested the presence of hydroxyl and/or secondary amine functionality. The UV absorptions at max 295, 287, 279, 230 nm in MeOH were characteristic to an indole chromophore. The COSY correlations (Figure 2) disclosed the ABCD system of the aromatic protons at δ_H_ 7.41/7.00 (Table 2), which is consistent with 2,3-disubstituted indole alkaloids. A side chain of an ethylene and a *N*-methyl was linked to C-3 of the indole as it was manifested by 2D NMR correlations. However, comparison of ^1^H NMR data of **18** with those of *N*-methyltryptamine (**17**) revealed that the ^1^H proton singlet at position 2 in **17** was replaced in **18** by a 2H singlet of a hydroxymethyl proton at δ_H_ 3.91 ppm. The ^13^C NMR spectroscopic and ESIMS data of **18** was thus consistent with a molecular formula C_12_H_16_N_2_O of 30 mass units more than that of **17** (C_11_H_14_N_2_). Furthermore, the HMBC correlation that was found from the methylene protons (δ_H_ 3.91, 2H, s) to C-2 and C-3 confirmed its C-2 location of the hydroxymethyl group. Finally, a full analysis of th COSY and HMBC spectral correlations (Figure 2) assigned the structure of compound **18** to be 3-(2-(methylamino) ethyl)-1H-indol-2-yl) methanol or 2-hydroxymethyl *N*-methyltryptamine, a new indole alkaloid.

Compounds **1**–**14**, **16**–**17** and **19**–**22**, which were also isolated from *J. pelargoniifolia* roots, were found to be identical to the previously reported natural products by comparison of their spectroscopic (IR, MS, and NMR) data and were identified as jatrophadiketone (**1**) was isolated from the roots of *J. curcas* [13], β-sitosterol (**2**) isolated from *J. curcas* seed kernels and from the methanolic extract of the root bark of *Calotropis gigantean* (Linn.), [14,15], curcuson D (**3**) and curcuson C (**4**) were isolated from *J. curcas* root extract [16], naringenin (**5**) was isolated from the root extract of *J. gossypifolia* [17,18], β-sitosterol glucoside (**6**) was isolated from the leave and twig extract of *J. curcas*, [19], spruceanol (**7**) was isolated from both the aerial extract of *J. divaricate* and the bark extract of *Aleurites moluccana* [20,21], propacin (**8**), cleomiscosin B (**9**), cleomiscosin A (**10**) compounds **8** and **10** were isolated from whole plant extracts of *J. gossypifolia*, while compound **9** was isolated from *Mallotus apelta* [22,23,24,25,26], apigenin (**11**) was identified in *J. gossypifolia* [4,27], uracil (**12**) was isolated from the leaves of *J. curcas* [28,29,30], cynaroside (**13**) was identified in *Scabiosa atropurpurea* aerial parts extract [31], linarin (**14**) was isolated from the extracts of aerial parts of both *Bupleurum chinense* and *Valeriana officinalis* [32,33], hovetricoside C (**16**) was separated from *Artocarpus tonkinensis* [34], *N*-methyltryptamine (**17**) was isolated from *Zanthoxylum arborescens* [35], *N*-methyltyramine (**19**) was isolated from a beer [36], and hordenine (**20**) was separated and identified from *Ephedra aphylla* that was growing in Egypt [37]. Compounds **21** (hordenine HCl) and **22** (*N*-methyltyramine HCl) were identical to the authentic samples that were purchased from Sigma-Aldrich (St Louis, MO, USA), and on the basis of their ^1^H NMR and TLC co-chromtographic data, they were isolated previously from *Ariocarpus kotschoubeyanus* [38].

### 2.3. Biological Activity

The alcoholic extract of *J. pelargoniifolia* was found to possess significant anti-inflammatory, analgesic, and antipyretic activities when it was tested on in vivo models in a dose-dependent manner [39]. This prompted us to extend the study of these activities on the isolated compounds. The anti-inflammatory, analgesic, antipyretic, and antioxidant activities for the compounds which have been isolated in good yields (**1**–**6**, **8**–**11**, **13**, **14**, **16**, **21**, and **22**) were thus evaluated for their analgesic, anti-inflammatory, antipyretic, and antioxidant activities using in vivo and in vitro models. The analgesic activities were assessed in mice via acetic acid-induced writhing, hot-plate, and tail-flick methods.

In the acetic acid-induced writhing method, compounds **1**, **4**, **6**, **9**, **11**, **13**, **14**, **16**, and **22** showed a dose-dependent analgesic activity by the reduction in the number of writhings. However, the diterpenoids (**1** and **4**), β-sitosterol glucoside (**6**), flavonoids (**5** and **13**)**,** and tryptamine HCl (**22**) exhibited the strongest analgesic activity by inhibiting writhing in mice (49.07–65.74% inhibition) at a dose of 10 mg/kg compared with the standard antinociceptive drug (indomethacin), which showed 72.68% reduction in the number of writhings at a concentration of 4 mg/kg (Table S9). The coumarinolignan (**8**) and hordenine HCl (**21**) did not show any inhibition either at 5 or at 10 mg/kg.

In the hot plate method, the thermal responses in the mice that were treated with selected compounds after half, one, and two hours were significantly reduced (*p* < 0.001). Especially in the mice that were treated with a dose 10 mg/kg of compounds **22, 10, 6, 4, 5, 13, 16**, and **14**, the antinociceptive effects were reduced by 78.57, 76.19, 74.46, 73.33, 65.95, 59.57, 53.48, and 34.88%, respectively (Table S10). While in the tail-flick method, the tested animals that were treated with 10 mg/kg of compounds **22, 1, 10, 4, 13, 16, 5, 6, 21, 3**, and **9** showed a significant (*p* < 0.001) reduction in antinociceptive activity (67.32, 67.21, 60.45, 57.59, 54.04, 53.59, 45.58, 40.30, 21.75, 20.39, and 10.87%, respectively) compared with indomethacin (95.61%), as depicted from Table S11. The obtained results confirmed that the strong analgesic activity that is exhibited by the roots of *J. pelargoniifolia* could be due to its bioactive compounds that may exert their analgesic activities through different CNS (Central Nervous System) mechanisms (peripheral and central). Therefore, further studies with purified compounds should be conducted in the future for further pharmacological and toxicological characterization in order to elucidate the mechanisms that are involved in the central analgesic effect of these compounds.

The anti-inflammatory activities of the major isolated compounds were evaluated by using the carrageenan-induced paw edema model in rats. It was found that the size of the edema was significantly reduced (*p* < 0.05–0.001) in the animals that were treated with the low doses of 5 and 10 mg/kg compared with the standard anti-inflammatory drug (phenylbutazone) at a high dose of 100 mg/kg. The rats that were treated with compounds **10**, **16**, **1**, **5**, **6**, **22**, **4**, **13**, **3**, **8**, **9**, **14**, and **21** exhibited a significant reduction in their hind paw edema in a dose-dependent manner. Therefore, at a dose of 5 mg/kg, the edema size was reduced by 20.44, 47.23, 17.95, 45.85, 33.97, 44.47, 13.53, 26.51, 7.18, 2.20, 5.52, 6.35, and 6.35, respectively, while at 10 mg/kg, the edema size was reduced by 64.91, 55.24, 54.94, 51.38, 51.10, 50.27, 49.17, 48.61, 13.53, 12.98, 10.22, 10.22, and 8.01%, respectively relative to that reduced by phenylbutazone at 100 mg/kg (69.06%) which was almost similar to that produced by a dose of 10 mg/kg of compound **10** (64.91%; Table S12). These anti-inflammatory results were almost compatible with those of the above mentioned antinociceptive activity for the tested compounds.

In addition, the isolated compounds from the *J. pelargoniifolia* roots were tested for their antipyretic activity against yeast-induced hyperthermia in mice. All tested compounds, which were administered at doses of 5 and 10 mg/kg, showed a considerable reduction in the rectal temperature of the hyper-thermic mice, ranging between 36.73 ± 0.13 °C and 38.56 ± 0.16 °C as compared with the hypothermic effect (36.33 ± 0.11 °C) resulting from indomethacin administration (Table S13). Moreover, compounds **5**, **6**, **10**, **13**, and **16** displayed about a 1 °C reduction in temperature less than that of the yeast-induced hyperthermia control (~ 38.8 °C) in the first 30 min. of the experiment.

The percentage inhibition ± SD of the nitric oxide-scavenging activity was determined for the selected compounds at concentrations of 20, 40, 60, 80, and 100 µg/mL, and the obtained results were compared with a standard antioxidant drug (ascorbic acid). Compounds **22**, **4**, **2**, **10**, **1**, **14**, **21**, **9**, **8**, **11**, **6**, **3**, **13**, **5**, and **16** exhibited significant free radical-scavenging potency when compared with the free radical-scavenging activity of a strong known antioxidant drug (ascorbic acid) 87.23 ± 0.98. The ability of the tested compounds to produce antioxidant effects was found to be concentration-dependent. At a 100 µg/mL dose, the % inhibition ± SD of the tested compounds were 77.60 ± 4.22, 77.36 ± 4.22, 76.83 ± 5.01, 75.26 ± 5.54, 71.66 ± 0.70, 70.36 ± 14.73, 67.67 ± 5.75, 63.67 ± 12.85, 63.67 ± 12.85, 57.00 ± 10.21, 56.54 ± 6.03, 38.30 ± 5.63, 33.06 ± 1.86, 27.00 ± 7.85, and 25.61 ± 5.18, respectively (Table S14). The significant antioxidant activity that was associated with the administration of *J. pelargoniifolia* roots was perhaps due to its content of several phenolic and polyphenolic compounds which play an important role in free radical-scavenging activity with less cytotoxicity.

It is important to mention here that in our previous study, which was carried out on the crude alcoholic extract of *J. pelargoniifolia* roots, we observed a significant anti-inflammatory activity and analgesic potency [39], likely resulting from the presence of cleomiscosin A, hovetricoside C, jatrophadiketone, naringenin, β-sitosterol glucoside, *N*-methyltyramine HCL, curcuson C, cynaroside, curcuson D, propacin, cleomiscosin B, linarin, and hordenine HCL in good yield. Undoubtedly, a synergistic effect between these bioactive constituents produces significant antinociceptive and anti-inflammatory effects. These results justify the use of this plant in folk medicine for the treatment of pain and several inflammatory conditions. Further study will be conducted on the pure isolated compounds to investigate the exact mechanisms underlying their promising biological activities.

Our study proved that *J. pelargoniifolia* roots can be considered as a source of several biologically- active compounds such as hordenine, which exhibited various biological activities like inhibiting melanogenesis in human melanocytes, increasing the respiratory and heart rates [40], the stimulation of gastrin release, inhibition of monoamine oxidase B, and antibacterial properties [41]. Furthermore, Chrisitine et al. reported that *N*-methyltyramine increases blood pressure in an anaesthetized rat, relaxes guinea pig ileum, and increases both the force and the rate of contraction of guinea-pig right atrium by inducing the release of noradrenaline [42]. Additionally, naringenin has been reported to have several pharmacological properties, including anti-dyslipidemic, anti-obesity and antidiabetic, and antifibrotic [43]. Moreover, cleomiscosin A showed strong anti-inflammatory activity and has analgesic and antipyretic potencies [44]. Curcuson C has been reported to have antipyretic activity in vivo [45].

## 3. Materials and Methods 

### 3.1. Chemicals and Analytical Instruments

The high-resolution electron spray ionization-mass spectrometry (HRESI-MS) analyses (Bruker, Bremen, Germany) were carried out on an Agilent Triple Quadrupole 6410 QQQ LC-MS mass spectrometer (Central Lab. College of Pharmacy, King Saud University (KSU)). The infra-red spectra were generally recorded in the potassium bromide pellets, unless otherwise specified, using the FTIR spectrophotometer (FT-IR Microscope Transmission, company, Waltham, MA, USA). The melting points were recorded by using a Mettler FP 80 Central Processor that was supplied with a Mettler FP 81 MBC Cell Apparatus. The spectral data for proton and carbon were measured by using Bruker AVANCE 700, 500, and 600 (College of Pharmacy, KSU and Department of Chemistry in TU Dortmund) (Bruker, Fallanden, Switzerland), resonating at either 700, 500, and 600 MHz for proton or at 125 MHz for carbon. The chemical shift values were expressed in ppm with respect to the internal standard tetramethyl silane (TMS) or residual solvent peak, and the coupling constants (*J*) were recorded in Hertz (Hz). The two-dimensional NMR experiments (COSY, HSQC, and HMBC) were performed using the standard Bruker program (Bruker, Fallanden, Switzerland). The silica gel 60/230–400 mesh (Qingdao Oceanic Chemical Co., Qingdao, China), RP C18 silica gel 40–63/230–400 mesh (Merck, Darmstade, Germany), and sephadex LH-20 with particle size 18–111 µm (GE Healthcare, Chicago, IL, USA) were used for column chromatography, while the silica gel and reversed phase 60 F254 (Merck, Germany) were used for thin-layer chromatography (TLC). The detection was achieved by using 10% H_2_SO_4_ in ethanol or ceric sulfate followed by heating. Alkaloids were tested with Mayer’s reagent, Hager’s reagent, and Dragendorff’s reagent. All of the solvents for analytical purposes (HPLC- and analytical-grade) and the drugs for biological investigation (sodium nitroprusside, sulphanilamide, λ-carrageenan, acetic acid, ascorbic acid, and phenylbutazone) were procured from Sigma Chemical Company (Sigma-Aldrich, St Louis, MO, USA), and the solvents were distilled prior to use. The preparative and semipreparative Shimadzu HPLC were performed, characterized by Rp-18 (ODS-80 TM, TSK, Tokyo, Japan), 10 μm PS, 30 cm L × 2.15 cm i.d. fitted with a guard column (10 μm PS, 7.5 cm L × 2.15 cm i.d.) (ODS-80 TM, TSK, Tokyo, Japan), and VP 250/10 NUCLEODUR C18 HTec, 6 μm PS, 25 cm L × 2 cm i.d., respectively which both used a PDA detector.

### 3.2. Plant Material

The roots of *J. pelargoniifolia* were harvested from Wadi Mojasas, Jazan district (South of Saudi Arabia) in September, 2015. The plant was authenticated by Dr. Jacob Thomas, a botanist of the Science College Herbarium, KSU, where a voucher specimen (#23064) was deposited. 

### 3.3. Animals

Male Wistar rats and white male Swiss albino mice with approximate body weights of 200 g and 20–25 g, respectively, were divided into groups of six animals. The animals were obtained from the Experimental Animal Care Center, College of Pharmacy, KSU. After a 7-day period in animal accommodation, they were divided into groups and were maintained at 12 h:12 h light-dark conditions at 55% humidity. Purina chow rat diet (UAR-Panlab, Barcelona, Spain) and drinking water were supplied to the animals ad libitum. The protocols for the present study were based on the recommendations of the Ethical Committee of the Experimental Animal Care Center of KSU (approval number CPR-7569).

### 3.4. Extraction, Fractionation, and Purification 

The air-dried powder of the *J. pelargoniifolia* roots (2.5 kg) was divided into two parts—A and B—and 2.5 kg of part A was subjected to solvent extraction, while the remaining 500 g of the root powder (part B) was exposed to the acid-base treatment. Part A was extracted by maceration with 80% ethanol (3 L × 5) for three successive days. This process was repeated until complete exhaustion of the plant material [46]. The alcoholic extract was then concentrated to dryness under reduced pressure at 40 °C using a rotary evaporator to give 270 g of the dried alcoholic extract. The dried alcoholic extract was suspended in H_2_O and was successively partitioned with petroleum ether, dichloromethane, ethyl acetate, and *n*-butanol (600–700 mL × 3) of each to obtain 13.3, 10.3, 5.1, and 33.6 g, respectively. 

A part of the petroleum ether fraction (12.8 g) was chromatographed over silica gel CC (Column Chromatography) using a gradient of petroleum ether/EtOAc followed by methanol (MeOH). The 100 mL fractions of each were collected and screened by TLC, and similar fractions were combined together to give six fractions (A–F). Fraction A which was eluted by 15% EtOAc in petroleum ether (609.8 mg) was further subjected to CC and was eluted by petroleum ether/acetone gradient elution, sub fraction A1 (188.9 mg) which was eluted by 6% acetone in petroleum ether was further purified by preparative HPLC gradient elution using acetonitrile: H_2_O: TFA) to yield 25.0 mg of compound **1**. Direct crystallization of fraction B, which was eluted by 20% EtOAc in petroleum ether, yielded 302.7 mg of compound **2**. Fractions C and D which were eluted with 30 and 40% EtOAc in petroleum ether, respectively, were crystallized with acetone to yield compounds **3** and **4** (14.3 and 25.4 mg, respectively). Additionally, fraction E (287.3 mg) which was eluted by 50% EtOAc was also crystallized from acetone to give 20.2 mg of compound **5**, while fraction F which was eluted with 40% MeOH in EtOAc yielded 330.4 mg of compound **6**, which was purified by crystallization with acetone.

The dichloromethane (DCM) fraction (9.8 g) was subjected to silica gel CC using a column that was packed by the wet method with petroleum ether. The polarity of the column was gradually increased by treating it with DCM, followed by MeOH to give 142 fractions, and similar fractions were pooled together depending on their TLC similarity. Fraction 48–64 which was eluted by 10% MeOH in DCM was concentrated (4.6 g) and was then subjected to repeated silica gel CC, followed by a preparative revered phase TLC using MeOH:H_2_O (3:1) as a solvent system, leading to the isolation of white crystals of compound **7** (7.5 mg). Moreover, subtractions that were obtained using 90% acetone in petroleum ether, 100% acetone, and 10% acetone in MeOH, followed by crystallization with MeOH, afforded compounds **8** (15.1 mg), **9** (15.9 mg), and **10** (16.4 mg), respectively. 

The EtOAC extract (4.6 g) was subjected to silica gel CC using a gradient of DCM/MeOH to give six fractions (I–IV). Fractions I which were eluted with 84% DCM afforded 17 mg of compound **11** after crystallization with MeOH. Fractions II which were eluted with 70% DCM afforded 8.6 mg of compound **12**. The fractions that were eluted with 35% and 40% MeOH in DCM (II and IV) were further purified by repeated acetone crystallization to give 14.9 and 13.2 mg of compounds **13** and **14**, respectively. Fractions V which were eluted with 45% MeOH were further subjected to CC using DCM/MeOH, followed by a semi-preparative HPLC (Rp-18) using MeOH:H_2_O:TFA as a solvent system afforded 8.6 mg white crystals of compound **15**. Finally, fractions VI which were eluted with 50% MeOH in DCM were subjected to further purification over sephadex LH-20 (using water and methanol as an eluent in the gradient mode). The subfraction VI–A, which was eluted by 20% H_2_O/MeOH was further purified over a reversed-phase column to give 12 mg of compound **16**. 

Furthermore, Part B was subjected to an acid-base treatment according to the Stas-Otto method I which was described by Mandhumitha and Fowsiya [47]. The crude alkaloidal fraction was subjected to silica columns using gradient elution with solvent system DCM/MeOH:NH_4_OH, resulting in five fractions. The first fraction which was eluted using 17% MeOH in DCM with an addition of 1% NH_4_OH was followed by further purification by reversed-phase semipreparative HPLC using MeOH:H_2_O:TFA to give compound **17** (6.3 mg). The second fraction which was separated by 20% MeOH in DCM to afford a subfraction, which was further purified by a semipreparative HPLC gradient elution using MeOH:H_2_O:TFA as a solvent system, afforded white needle crystals of compound **18** (9.4 mg). The third and fourth fractions which were eluted by 23% and 26% MeOH in DCM, followed by an addition of a few drops of NH_4_OH, afforded 6.8 and 8.8 mg of compounds **19** and **20**, respectively. The fifth fraction was eluted by 60% MeOH in DCM with an addition of a few NH_4_OH drops to yield 86.7 mg of a mixture of two compounds, which were subjected to further purification using the reversed-phase semipreparative HPLC in gradient mode with MeOH:H_2_O:TFA as the mobile phase, resulting in the production of the white crystals of compounds **21** and **22** (20.3 and 21.5 mg), respectively.

6-hydroxy-8-methoxycoumarin-7-*O*-β-d-glycopyranoside (compound **15**): White crystals; m.p. 219–220 °C; UV (MeOH) λ_max_ nm 325 and 250; IR (KBr) νmax (cm^−1^): 3349, 1719, 1625, 1520, 1465, 829; ^1^H NMR, ^13^C NMR, and HMBC data, see Table 1 and Figure 2; HRESIMS (positive) *m*/*z* 371.0900 [M + H]^+^ (calculated for C_16_H_18_O_10_, 371.097825).

3-(2-(methylamino)ethyl)-1H-indol-2-yl)methanol (compound 18): White needle crystals; m.p. 179–189 °C; UV (MeOH) λ_max_ nm: 295, 287, 279, 230; IR (KBr) ν_max_ (cm^−1^): 3309,1140, 1120, 1105, 1011, 855. ^1^H NMR, ^13^C NMR, and HMBC data, see Table 2 and Figure 2; HRESIMS (positive) *m*/*z* 205.1293 [M + H]^+^ (calculated for C_12_H_16_N_2_O, 205.134088).

### 3.5. Antinociceptive Activity Test

#### 3.5.1. Hot-plate Method

The hot-plate method that was described by Turner was used to determine the antinociceptive activity of the compounds that were isolated from the *J. pelargoniifolia* root [48].

#### 3.5.2. Acetic Acid-induced Writhing in Mice Test

The method of Koster et al. was used to evaluate the analgesic effect of the pure compounds that were isolated from the *J. pelargoniifolia* root [49].

#### 3.5.3. Tail-Flick Method

Acute nociception was induced using the tail-flick apparatus (Tail flick Apparatus Harvard), following the method that was recommended by D’amour and Smith [50].

### 3.6. Anti-Inflammatory Activity Test

#### Carrageenan-Induced Edema in the Rat Paw Method

The method that was described by Winter et al. was used to evaluate the anti-inflammatory potency of the isolated compounds [51].

### 3.7. Antipyretic Activity Screening

#### Yeast-Induced Hyperthermia in Rats

Hyperthermia was induced in the mice followed by the administration of the isolated compounds, and their hypothermic activity was determined by applying the method described by Loux [52].

### 3.8. Antioxidant Effect 

#### Nitric Oxide Radical-Scavenging Assay

This assay was carried out according to the procedure that was described by Green et al. [53].

### 3.9. Statistical Analysis

The values in the tables are given as mean ± SE. The data were analyzed by using one-way analysis of variance (ANOVA) followed by the Student’s t-test. Values with *p* < 0.05 were considered significant.

## 4. Conclusions

The wide traditional use of *Jatropha* species as anti-inflammatory and analgesics has prompted us to investigate the chemistry and bioactivity of *J. pelargoniifolia* growing in Saudi Arabia. The phytochemical study of the plant roots resulted in the isolation of six terpenoids, five flavonoids, three coumarinolignans, two tryptamines, and two tyramines (including their HCl salts), a coumarin, and a pyrimidine. The new compounds were identified as 6-hydroxy-8-methoxy coumarin-7-*O*-β-d-glycopyranoside and 2-hydroxymethyl-*N*-methyltryptamine. To the best of our knowledge, hovetricoside C and *N*-methyltryptamine were isolated herein from the Euphorbiaceae family for the first time, while cleomiscosin B, hordenine, and *N*-methyltyramine with their salts, cynaroside, and linarin were characterized in the *Jatropha* species for the first time.

On the basis of the significant anti-inflammatory, analgesic, antipyretic, and antioxidant activities that were observed in the experimental animals for the alcoholic extract of *J. pelargoniifolia*, fifteen of the adequately isolated compounds were consequently biologically evaluated. Eleven of these compounds exhibited strong analgesic activity. Twelve out of the fifteen compounds succeeded to reduce the chemically-induced inflammatory marker in the animals in a dose-dependent manner. Moreover, five of the compounds demonstrated an anti-pyretic effect by a reduction about a 1 °C in an induced hyperthermia model. The isolated compounds also exhibited varying degrees of nitric oxide-scavenging activity. The significant antioxidant activity that was associated with the administration the extract of *J. pelargoniifolia* roots was thus perhaps due to its phenolic content such as flavonoid, coumarins, and coumarinolignans. The synergistic effect between these bioactive constituents might explain the significant antinociceptive and anti-inflammatory effect of the alcoholic extract of *J. pelargoniifolia* roots and may scientifically justify the use of this plant in folk medicine for the treatment of pain and several inflammatory conditions.

## Figures and Tables

**Table 1:** The ^1^H (600 MHz, δ in ppm, *J* in Hz) and ^13^C NMR (125MHz, δ in ppm) spectral data for compound **15** in deuterated methanol (CD_3_OD).

Position	δ_H_	δ_C_
2	-	163.5
3	6.26 (d, *J* = 9.5 Hz, 1H)	116.2
4	7.88 (d, *J* = 9.5 Hz, 1H)	146.5
5	7.00 (s, 1H)	106.1
6	-	145.7
7	-	133.2
8	-	147.5
4a	-	112.7
8a	-	144.4
1′	4.99 (d, *J* = 7.8 Hz, 1H)	106.2
2′	3.57 (dd, *J* = 9.4, 9.4, 1H)	75.5
3′	3.46 (d, *J* = 1.9, 1H)	77.8
* 4′	3.47 (brs, 1H)	71.0
* 5′	3.30 (brs, 1H)	78.5
6′	3.72 (d, *J* = 4.9, 1H) 3.80 (d, *J* = 2.4, 1H)	62.2
OCH_3_-8	3.91 (s, 3H)	57.0
OH-6	10.53	-

* Overlapped with solvent signal.

**Table 2:** The ^1^H (700 MHz, δ in ppm, *J* in Hz) and ^13^C NMR (125 MHz, δ in ppm) spectral data for compound **18** in deuterated methanol (CD_3_OD).

Position	δ_H_	δ_C_
2	-	128.0
3	-	107.2
3a	-	130.6
4	7.29 (d, *J* = 7.8 Hz, 1H)	112.0
5	7.07 (t, *J* = 7.8 Hz, 1H)	122.4
6	7.00 (t, *J* = 7.8 Hz, 1H)	120.0
7	7.41 (d, *J* = 7.8 Hz, 1H)	118.6
7a	-	138.1
αCH_2_	3.10 (t, *J* = 5.8 Hz, 2H)	54.1
βCH_2_	3.00 (t, *J* = 5.8 Hz, 2H)	21.5
CH_3_	2.69 (s, 3H)	44.9
CH_2_OH	3.91 (s, 2H)	52.9

## Supplementary Materials

The following are available online. Figures S1–S4: ^1^H-, ^13^C-NMR, COSY and HMBC spectra of compound **15**, Figures S5–S8: ^1^H-, ^13^C-NMR, HMBC and HSQC spectra of compound **18**, Tables S9–S11: Analgesic effect of isolated compounds by using acetic acid-induced writhing, hot plate and tail flick methods in mice, Table S12: Effect of isolated compounds on carrageenan-induced paw edema in albino rats, Table S13: Effect of isolated compounds on yeast-induced hyperthermia in mice, Table S14: % Inhibition of nitric oxide scavenging activity for isolated compounds at different concentrations.

## Appendix A

Supplementary data associated with ^1^H NMR, ^13^C NMR, COSY, and HMBC of compounds 15 and 18 are available in Supplementary Information. In addition, Tables for all of the biological test results are provided.
