# Supplementary material for: The Phytochemical and Biological Investigation of Jatropha pelargoniifolia Root Native to the Kingdom of Saudi Arabia
Source: Molecules. 2018 Jul 28;23(8):1892. doi: 10.3390/molecules23081892 (PMC6222854; doi:10.3390/molecules23081892)
Supplement: Supplementary file 1 [file molecules-23-01892-s001.pdf]

Article

The Phytochemical and Biological Investigation of *Jatropha pelargoniifolia* Root Native to the Kingdom of Saudi Arabia

Hanan Y. Aati [1], Ali A. El-Gamal [1,2,*], Oliver Kayser [3] and Atallah F. Ahmed [1,2]

1 Department of Pharmacognosy, Faculty of Pharmacy, King Saud University, Riyadh 11451, Saudi Arabia; hati@ksu.edu.sa (H.Y.A.); afahmed@ksu.edu.sa (A.F.A.)
2 Department of Pharmacognosy, College of Pharmacy, Mansoura University, El-Mansoura 35516, Egypt
3 TU Dortmund University, Technical Biochemistry, Emil-Figge-Strasse 66, D-44227 Dortmund, Germany; oliver.kayser@tu-dortmund.de
* Correspondence: aelgamel@ksu.edu.sa; Tel.: +966-1146-77259

Received: 29 May 2018; Accepted: 25 July 2018; Published: date

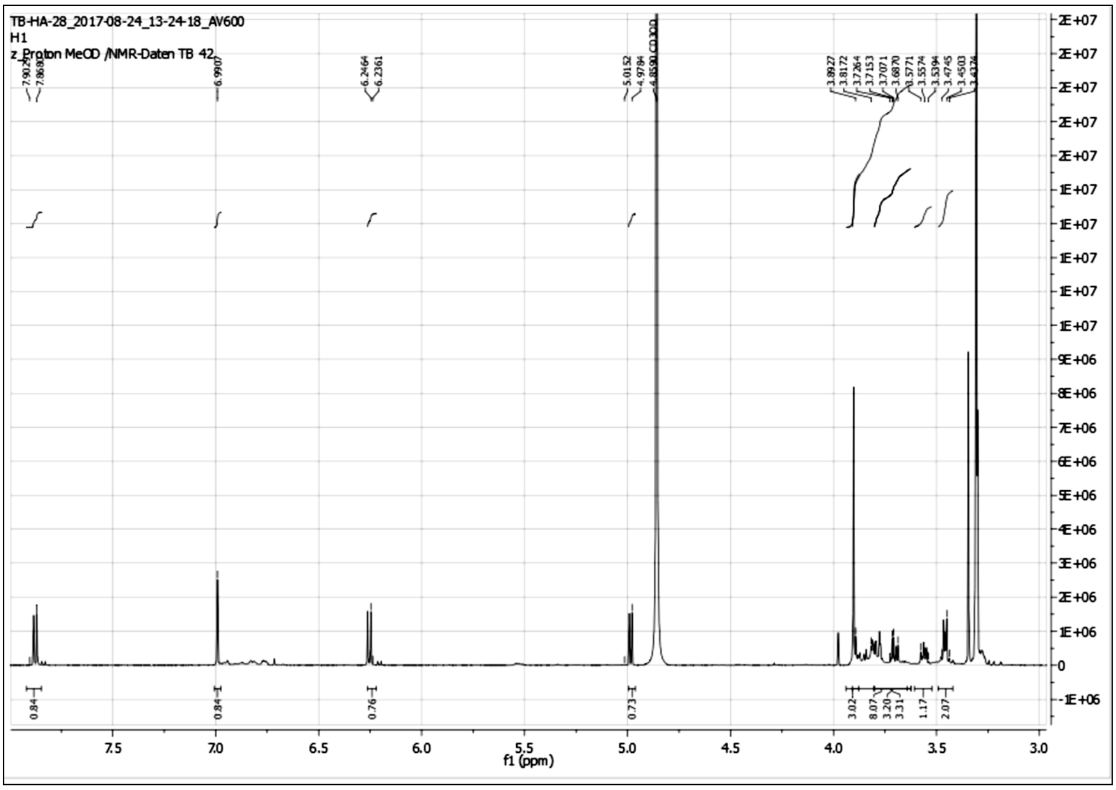

Figure S1. ^{1}H NMR spectrum for compound **15** in CD_3OD (600 MHz).

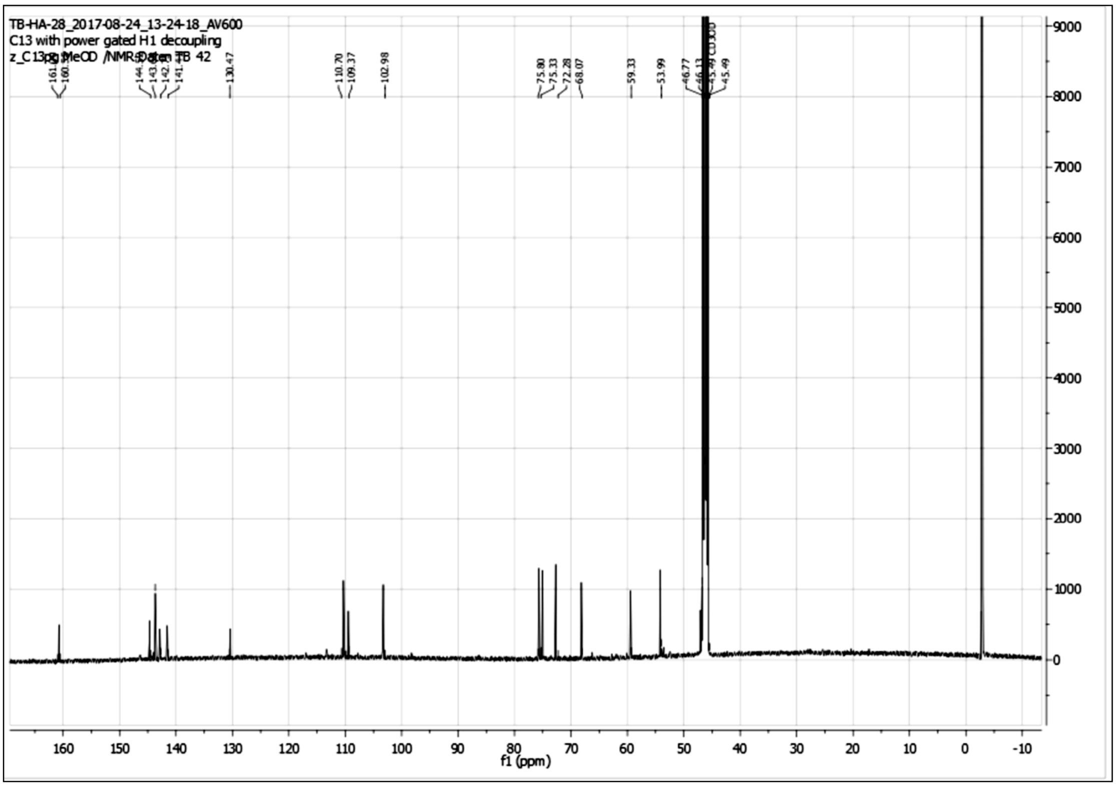

Figure S2. ^{13}C NMR spectrum for compound **15** in CD_3OD (125 MHz).

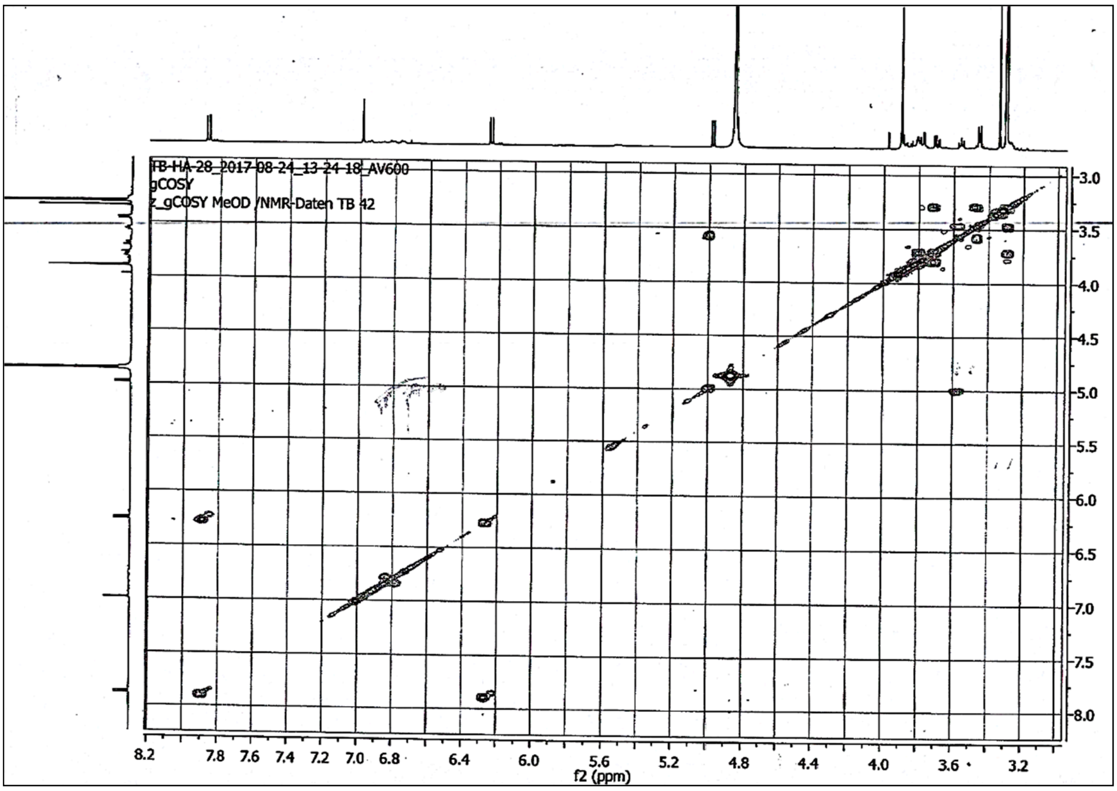

Figure S3. COSY spectrum for compound **15.**

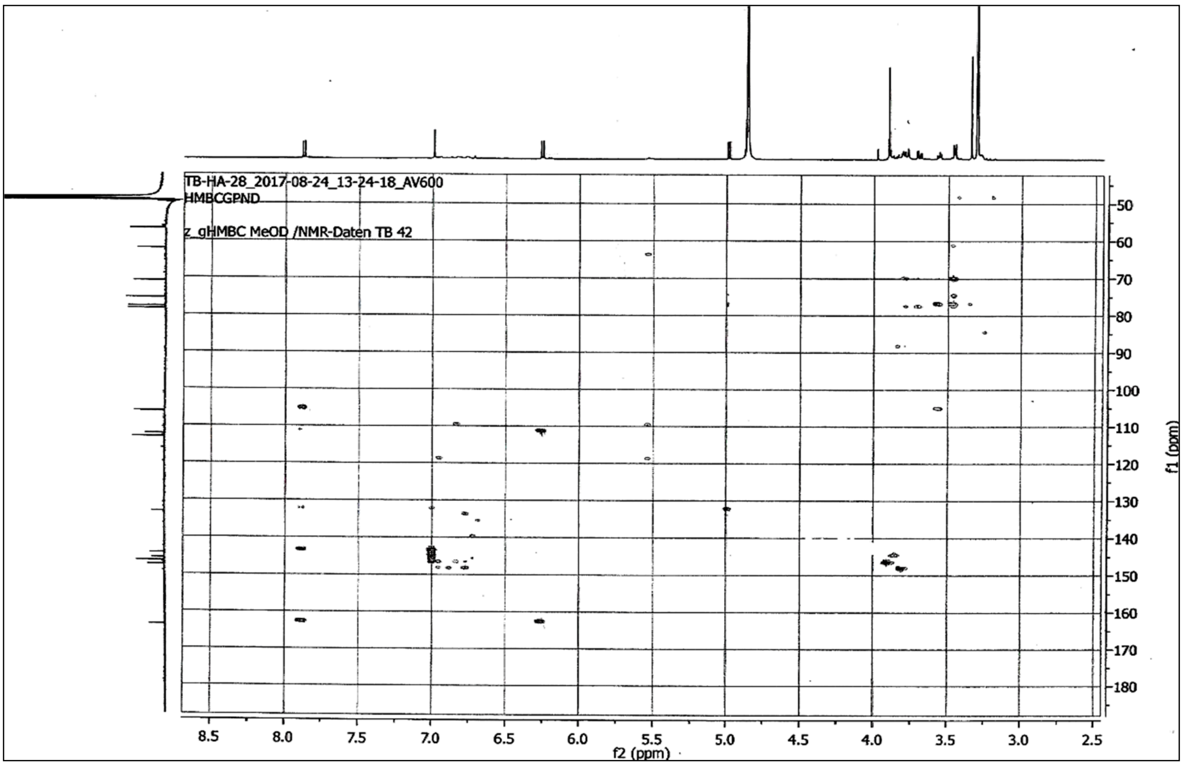

Figure S4. HMBC spectrum for compound **15.**

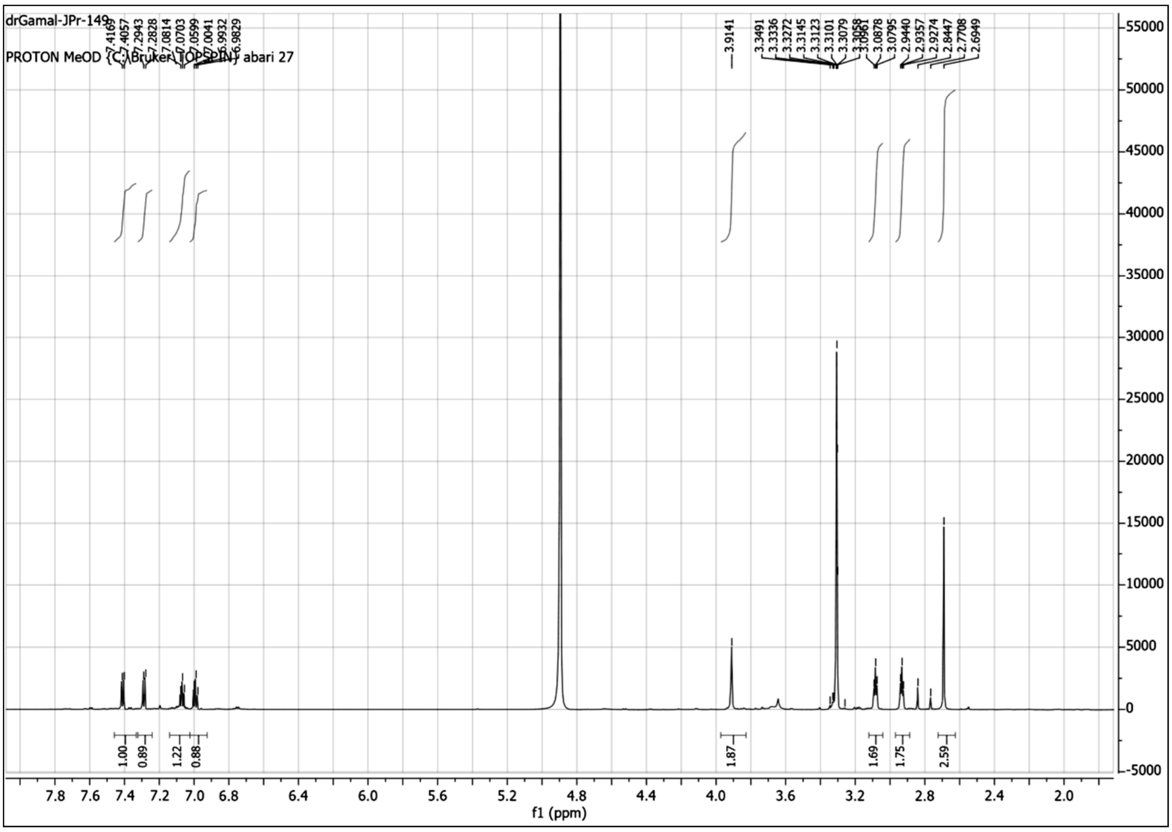

Figure S5. ^{1}HNMR spectrum for compound **18** in CD_3OD (700 MHz).

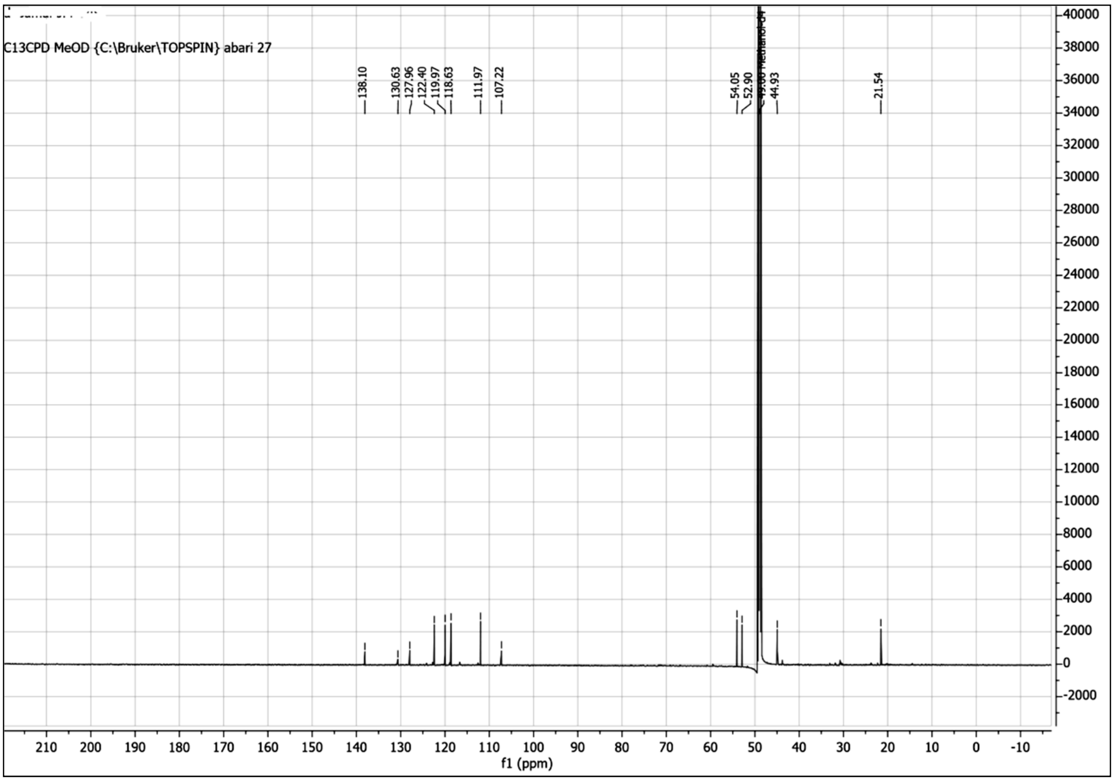

Figure S6. ^{13}CNMR spectrum for compound **18** in CD_3OD (125 MHz).

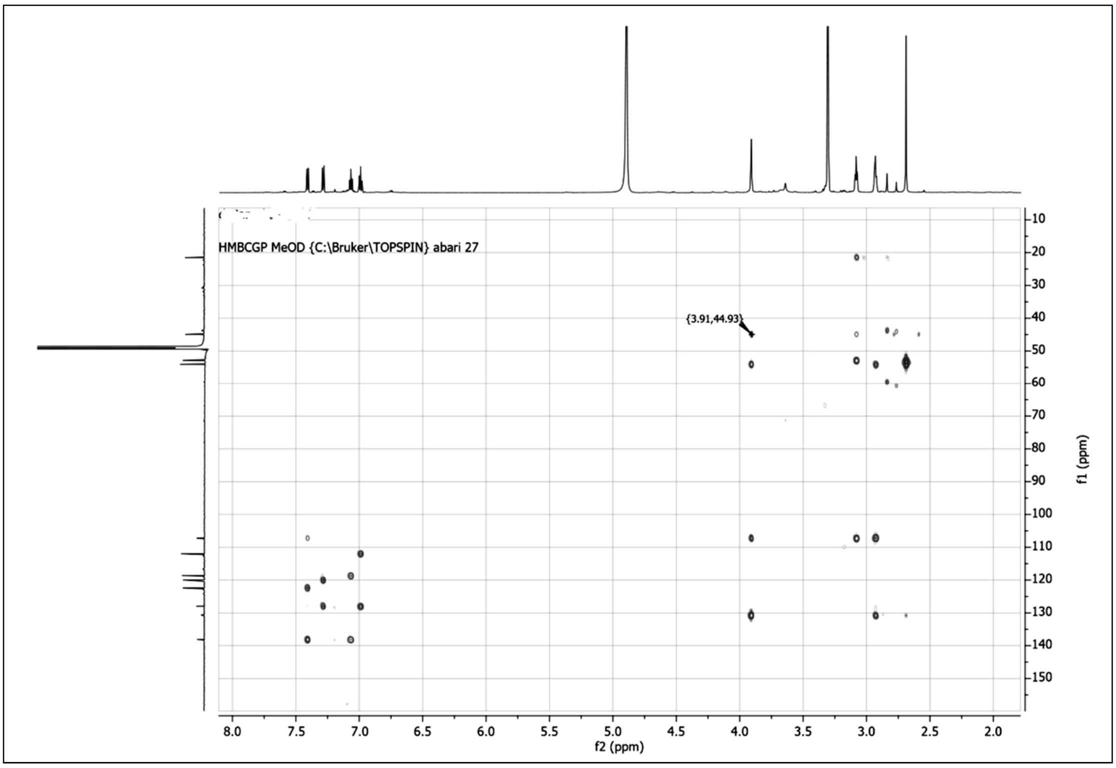

Figure S7. HMBC spectrum for compound **18**.

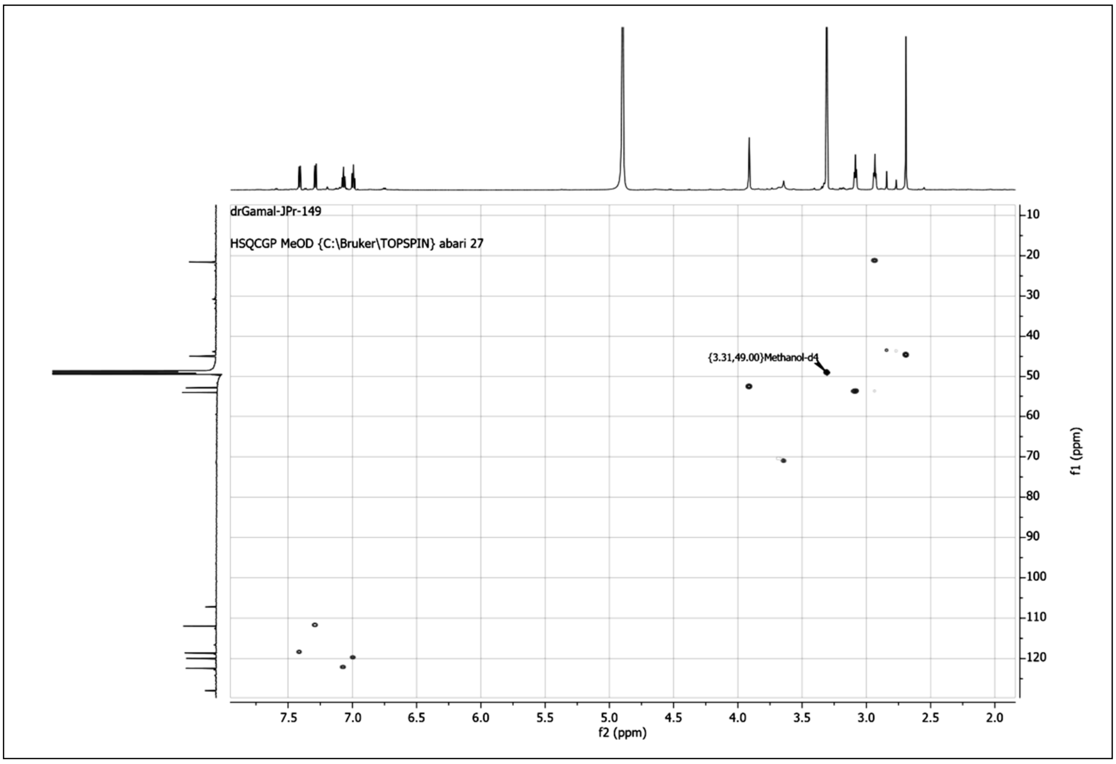

Figure S8. HSQC spectrum for compound **18.**

Table S9. Analgesic effect of the isolated compounds by using acetic acid-induced writhing in mice.

**Treatments (n = 6)**	**Dose (mg/kg)**	**Number of writhing in 20 min**	**% Inhibition**
Control (20% acetic acid)	0.1 mL	36.00 ± 1.15	-
No-2	5	34.33 ± 1.28	4.62
No-2	10	34.50 ± 1.31	4.16
No-6	5	24.66 ± 1.20 ***	31.48
No-6	10	17.00 ± 0.85 ***	52.77
No-5	5	18.16 ± 0.47 ***	49.53
No-5	10	13.33 ± 0.49 ***	62.96
No-11	5	36.66 ± 0.66	1.85
No-11	10	32.50 ± 0.92 *	9.72
No-14	5	30.83 ± 1.13 *	14.35
No-14	10	27.50 ± 0.76 ***	23.61
No-13	5	21.16 ± 0.98 ***	41.20
No-13	10	15.00 ± 0.57 ***	58.33
No-16	5	26.83 ± 1.04 ***	25.46
No-16	10	25.33 ± 0.66 ***	29.62
No-3	5	35.66 ± 1.70	•
No-3	10	32.33 ± 1.42	10.18
No-4	5	23.16 ± 1.01 ***	35.64
No-4	10	18.33 ± 0.88 ***	49.07
No-1	5	24.33 ± 1.30 ***	32.40
No-1	10	18.16 ± 0.94 ***	49.53
No-8	5	37.83 ± 1.01	•
No-8	10	37.16 ± 1.32	•
No-9	5	34.00 ± 1.36	5.55
No-9	10	30.00 ± 1.06 **	16.16
No-10	5	26.50 ± 0.88 ***	26.38
No-10	10	24.16 ± 0.94 ***	32.87
No-21	5	38.00 ± 0.96	•
No-21	10	37.00 ± 0.96	•
No-22	5	24.00 ± 1.48 ***	33.33
No-22	10	12.33 ± 0.55 ***	65.74
**Indomethacin**	4	9.83 ± 0.60 ***	72.68

All values represent mean ± SEM.* p <0.05, ** p <0.01, *** p <0.001; ANOVA, followed by Dunnett's multiple comparison test, compounds, and indomethacin groups compared with acetic acid groups.

•% inhibition is very low or no effect

Table S10. Analgesic effect of the isolated compounds by using the hot plate method in mice.

Treatments	Dose mg/kg	Reaction time (sec.) pre-drug	Reaction time (sec.) post-drug					
			30 min	% Change	60 min	% Change	120 min	% Change
No-2	5	7.16 ± 0.30	6.83 ± 0.30	4.65	7.66 ± 0.33	6.97	7.66 ± 0.33	6.97
No-2	10	7.00 ± 0.36	7.83 ± 0.30	9.30	7.66 ± 0.33	6.97	8.33 ± 0.42 *	16.27
No-6	5	8.00 ± 0.36	8.66 ± 0.33	8.33	10.16 ± 0.30 ***	27.08	11.66 ± 0.42 ***	45.83
No-6	10	7.83 ± 0.30	11.00 ± 0.36 ***	40.25	12.66 ± 0.33 ***	61.70	13.66 ± 0.42 ***	74.46
No-5	5	7.83 ± 0.30	9.33 ± 0.49*	19.14	10.50 ± 0.42 ***	30.04	11.33 ± 0.33 ***	44.68
No-5	10	7.66 ± 0.49	11.00 ± 0.36 ***	40.25	12.66 ± 0.33 ***	61.70	13.00 ± 0.26 ***	65.95
No-11	5	7.83 ± 0.30	7.33 ± 0.42	•	7.66 ± 0.33	•	8.33 ± 0.33	6.38
No-11	10	7.33 ± 0.33	8.00 ± 0.36	•	7.83 ± 0.30	•	8.83 ± 0.30**	20.45
No-14	5	7.50 ± 0.42	7.83 ± 0.30	4.44	8.16 ± 0.30	8.88	9.16 ± 0.47 *	22.22
No-14	10	7.16 ± 0.30	7.83 ± 0.30	9.30	8.33 ± 0.33 *	16.27	9.66 ± 0.42 ***	34.88
No-13	5	8.00 ± 0.36	9.33 ± 0.33*	16.66	10.66 ± 0.42 ***	33.33	11.00 ± 0.36 ***	37.50
No-13	10	7.83 ± 0.30	11.00 ± 0.36 ***	40.25	11.66 ± 0.42 ***	48.93	12.50 ± 0.61 ***	59.57
No-16	5	7.16 ± 0.30	8.00 ± 0.36	11.62	9.00 ± 0.36 **	25.58	10.16 ± 0.30 ***	41.86
No-16	10	7.16 ± 0.30	8.66 ± 0.42*	20.93	10.83 ± 0.40 ***	51.16	11.00 ± 0.36 ***	53.48
No-3	5	7.00 ± 0.36	7.33 ± 0.33	4.76	7.16 ± 0.30	2.38	8.33 ± 0.42 *	19.04
No-3	10	7.00 ± 0.36	7.33 ± 0.33	4.76	7.83 ± 0.30	11.90	9.00 ± 0.36 **	28.57
No-4	5	7.16 ± 0.30	7.66 ± 0.33	6.97	8.50 ± 0.42 *	18.60	9.83 ± 0.47 ***	37.20
No-4	10	7.50 ± 0.42	10.00 ± 0.57 **	33.33	10.50 ± 0.42 ***	40.00	13.00 ± 0.36 ***	73.33
No-1	5	7.66 ± 0.33	7.50 ± 0.42	•	7.66 ± 0.33	•	8.00 ± 0.44	4.34
No-1	10	7.50 ± 0.42	7.66 ± 0.49	2.22	7.83 ± 0.40	4.44	8.33 ± 0.49	11.11
No-8	5	7.00 ± 0.36	7.33 ± 0.33	4.76	7.50 ± 0.42	7.14	8.16 ± 0.40	16.16
No-8	10	7.16 ± 0.30	7.66 ± 0.49	6.97	8.16 ± 0.30 *	13.95	9.00 ± 0.36 **	25.58
No-9	5	6.83 ± 0.30	7.00 ± 0.36	2.43	7.83 ± 0.30 *	14.63	8.16 ± 0.40 *	19.51
No-9	10	7.66 ± 0.33	7.83 ± 0.30	2.17	8.66 ± 0.42 *	13.04	9.83 ± 0.47 **	28.26
No-10	5	7.16 ± 0.30	8.00 ± 0.25	11.62	8.66 ± 0.42 *	20.93	10.16 ± 0.30 ***	41.86
No-10	10	7.00 ± 0.36	9.00 ± 0.25**	28.57	10.50 ± 0.34 ***	50.00	12.33 ± 0.49 ***	76.19
No-21	5	6.83 ± 0.30	7.00 ± 0.36	2.43	7.16 ± 0.30	4.87	8.00 ± 0.36	17.07
No-21	10	7.00 ± 0.36	7.66 ± 0.33	8.52	7.50 ± 0.42	7.14	7.83 ± 0.40	11.90
No-22	5	7.00 ± 0.36	7.83 ± 0.30	11.90	8.66 ± 0.42*	23.89	10.33 ± 0.42 ***	47.61
No-22	10	7.00 ± 0.36	8.50 ± 0.34 *	21.42	9.16 ± 0.40 **	30.95	12.50 ± 0.42 ***	78.57
**Indomethacin**	4	7.16 ± 0.30	10.16 ± 0.30 ***	41.86	12.83 ± 0.40 ***	79.06	14.66 ± 0.33 ***	104.65

All values represent mean ± SEM.* p <0.05, ** p <0.01, *** p <0.001; ANOVA, followed by Dunnett's multiple comparison test, post-drug compared with pre-drug group. •% inhibition is very low or no effect.

Table S11. Analgesic effect of the isolated compounds by using the tail flick method in mice.

Treatments	Dose mg/kg	Reaction time (s) pre-drug	Reaction time (s) post-drug					
			30 min	% Change	60 min	% Change	120 min	% Change
No-2	5	5.20 ± 0.19	5.11 ± 0.18	•	5.30 ± 0.23	•	5.16 ± 0.12	•
No-2	10	5.10 ± 0.27	5.13 ± 0.12	•	5.28 ± 0.20	3.59	5.08 ± 0.08	•
No-6	5	5.15 ± 0.13	5.45 ± 0.13	5.82	5.88 ± 0.13 **	14.23	5.96 ± 0.15 **	15.85
No-6	10	5.41 ± 0.16	5.98 ± 0.10 *	10.45	6.70 ± 0.10 ***	23.69	7.60 ± 0.37 ***	40.30
No-5	5	4.93 ± 0.13	6.06 ± 0.07 ***	22.97	6.48 ± 0.16 ***	31.41	7.00 ± 0.09 ***	41.89
No-5	10	5.28 ± 0.12	6.78 ± 0.11 ***	28.39	7.33 ± 0.15 ***	38.80	7.85 ± 0.17 ***	45.58
No-11	5	5.20 ± 0.15	5.01 ± 0.13	•	5.11 ± 0.10	•	5.25 ± 0.09	•
No-11	10	5.25 ± 0.19	5.11 ± 0.13	•	5.05 ± 0.11	•	5.26 ± 0.11	•
No-14	5	4.93 ± 0.12	4.86 ± 0.08	•	4.98 ± 0.08	•	5.18 ± 0.11	•
No-14	10	5.05 ± 0.13	5.13 ± 0.14	•	5.38 ± 0.11	6.60	5.76 ± 0.18 *	14.19
No-13	5	4.96 ± 0.11	5.30 ± 0.10	6.71	6.15 ± 0.11 ***	23.82	6.53 ± 0.18 ***	31.54
No-13	10	5.15 ± 0.10	6.01 ± 0.16 ***	16.82	6.95 ± 0.19 ***	34.95	7.93 ± 0.14 ***	54.04
No-16	5	5.21 ± 0.13	5.96 ± 0.12 **	14.37	7.26 ± 0.56 **	39.55	7.00 ± 0.23 ***	34.18
No-16	10	5.10 ± 0.11	6.80 ± 0.14 ***	33.33	7.25 ± 0.13 ***	42.15	7.83 ± 0.17 ***	53.59
No-3	5	5.10 ± 0.12	5.13 ± 0.10	*	5.35 ± 0.15	4.90	5.76 ± 0.14 **	13.07
No-3	10	5.06 ± 0.14	5.46 ± 0.21	7.89	5.41 ± 0.18	6.90	6.10 ± 0.12 ***	20.39
No-4	5	5.11 ± 0.09	6.01 ± 0.20 **	17.58	6.63 ± 0.17 ***	29.64	7.18 ± 0.14 ***	40.39
No-4	10	5.26 ± 0.19	6.70 ± 0.26 **	27.21	7.68 ± 0.15 ***	45.88	8.30 ± 0.23 ***	57.59
No-1	5	4.83 ± 0.25	6.03 ± 0.13 **	28.82	6.31 ± 0.15 ***	30.68	7.00 ± 0.09 ***	44.82
No-1	10	5.08 ± 0.12	7.21 ± 0.14 ***	41.96	7.86 ± 0.17 ***	54.75	8.50 ± 0.26 ***	67.21
No-8	5	4.86 ± 0.12	5.08 ± 0.11	4.45	5.36 ± 0.13 *	10.27	5.50 ± 0.16 *	13.01
No-8	10	5.15 ± 0.19	5.16 ± 0.09	•	5.41 ± 0.12	5.17	5.75 ± 0.09 *	11.65
No-9	5	4.98 ± 0.14	5.16 ± 0.14	3.67	5.30 ± 0.10	6.35	5.85 ± 0.12 ***	17.39
No-9	10	5.51 ± 0.17	5.86 ± 0.11	6.34	6.05 ± 0.12 *	9.66	6.11 ± 0.09 ***	10.87
No-10	5	5.05 ± 0.13	5.88 ± 0.15 **	16.50	6.68 ± 0.11 ***	32.34	7.10 ± 0.08 ***	40.59
No-10	10	5.10 ± 0.14	6.73 ± 0.16 ***	32.02	7.15 ± 0.25 ***	40.19	8.18 ± 0.11 ***	60.45
No-21	5	5.03 ± 0.25	5.18 ± 0.18	2.98	5.26 ± 0.14	4.63	5.15 ± 0.18	9.60
No-21	10	5.13 ± 0.13	5.23 ± 0.12	•	5.96 ± 0.08 ***	16.23	6.25 ± 0.08 ***	21.75
No-22	5	5.46 ± 0.19	6.63 ± 0.28 **	21.34	7.00 ± 0.25 ***	28.04	7.56 ± 0.12 ***	38.10
No-22	10	5.05 ± 0.14	7.06 ± 0.24 ***	39.93	7.98 ± 0.10 ***	58.08	8.45 ± 0.16 ***	67.32
**Indomethacin**	4	5.31 ± 0.15	7.65 ± 0.24 ***	43.48	8.05 ± 0.25 ***	51.41	10.40 ± 0.42 ***	95.61

All values represent mean ± SEM.**p* <0.05, ** *p* <0.01, ****p* <0.001; ANOVA, followed by Dunnett's multiple comparison test, the post-drug group was compared with the pre-drug group. •% inhibition is very low or no effect.

Table S12. Effect of the isolated compounds on carrageenan-induced paw edema in albino rats.

Treatments	Dose mg/kg	Before carrageenan	3 h after carrageenan	Net	% Inhibition
**Only carrageenan**	0.2 mL	1.06 ± 0.02	1.66 ± 0.01***	0.60 ± 0.007	-
No-2	5	1.06 ± 0.02	1.67 ± 0.0***	0.60 ± 0.008	•
No-2	10	1.05 ± 0.02	1.64 ± 0.01***	0.59 ± 0.04	•
No-6	5	1.08 ± 0.02	1.48 ± 0.02***	0.39 ± 0.009***	33.97
No-6	10	1.07 ± 0.02	1.36 ± 0.02***	0.29 ± 0.01***	51.10
No-5	5	1.08 ± 0.02	1.48 ± 0.02***	0.32 ± 0.02***	45.85
No-5	10	1.07 ± 0.01	1.36 ± 0.02***	0.29 ± 0.01***	51.38
No-11	5	1.04 ± 0.02	1.65 ± 0.01***	0.60 ± 0.02	•
No-11	10	1.07 ± 0.02	1.67 ± 0.01***	0.60 ± 0.001	•
No-14	5	1.10 ± 0.01	1.66 ± 0.02***	0.56 ± 0.02	6.35
No-14	10	1.09 ± 0.007	1.64 ± 0.01***	0.54 ± 0.02*	10.22
No-13	5	1.06 ± 0.02	1.50 ± 0.02***	0.44 ± 0.02***	26.51
No-13	10	1.02 ± 0.03	1.33 ± 0.03***	0.31 ± 0.009***	48.61
No-16	5	1.06 ± 0.002	1.50 ± 0.002***	0.31 ± 0.003***	47.23
No-16	10	1.02 ± 0.03	1.33 ± 0.03***	0.27 ± 0.007***	55.24
No-3	5	1.09 ± 0.008	1.59 ± 0.01***	0.56 ± 0.01*	7.18
No-3	10	1.04 ± 0.03	1.56 ± 0.02***	0.52 ± 0.02**	13.53
No-4	5	1.07 ± 0.02	1.60 ± 0.02***	0.52 ± 0.01**	13.53
No-4	10	1.08 ± 0.01	1.41 ± 0.01***	0.30 ± 0.004***	49.17
No-1	5	1.05 ± 0.03	1.54 ± 0.02***	0.49 ± 0.04*	17.95
No-1	10	1.09 ± 0.01	1.36 ± 0.01***	0.27 ± 0.009***	54.94
No-8	5	1.09 ± 0.009	1.68 ± 0.001***	0.59 ± 0.01	2.20
No-8	10	1.13 ± 0.01	1.65 ± 0.02***	0.52 ± 0.03*	12.98
No-9	5	1.04 ± 0.02	1.61 ± 0.01***	0.57 ± 0.01	5.52
No-9	10	1.08 ± 0.02	1.62 ± 0.01***	0.54 ± 0.01**	10.22
No-10	5	1.00 ± 0.03	1.48 ± 0.01***	0.48 ± 0.03**	20.44
No-10	10	1.10 ± 0.006	1.31 ± 0.01***	0.21 ± 0.01***	64.91
No-21	5	1.09 ± 0.03	1.66 ± 0.009***	0.56 ± 0.01*	6.35
No-21	10	1.06 ± 0.02	1.62 ± 0.02***	0.55 ± 0.01*	8.01
No-22	5	1.10 ± 0.007	1.43 ± 0.02***	0.33 ± 0.007***	44.47
No-22	10	1.08 ± 0.01	1.38 ± 0.009***	0.30 ± 0.008***	50.27
**Phenylbutazone**	100	1.03 ± 0.02	1.22 ± 0.02***	0.18 ± 0.007***	69.06

All values represent mean ± SEM.*p <0.05, **p <0.01, ***p <0.001; ANOVA, followed by Dunnett's multiple comparison test. The compound and phenylbutazone groups were compared with the carrageenan groups. •% inhibition is very low or no effect.

Table S13. Effect of the isolated compounds on yeast-induced hyperthermia in mice.

Treatments	Dose mg/kg	Normal Rector Temperature	Rectal Temperature after Yeast Administration 20 ml/kg of 20%	Rectal temperature after drug administration 20 ml/kg of 20%		
				30 min	60 min	120 min
No-2	5	35.06 ± 0.12	38.83 ± 0.10 ***	38.80 ± 0.12	38.73 ± 0.14	38.20 ± 0.12 *
No-2	10	35.16 ± 0.12	38.65 ± 0.12 ***	38.51 ± 0.15	38.45 ± 0.13	38.08 ± 0.12 *
No-6	5	35.20 ± 0.11	38.76 ± 0.14 ***	38.25 ± 0.08 *	38.26 ± 0.12 *	37.78 ± 0.11 ***
No-6	10	35.16 ± 0.10	38.81 ± 0.12 ***	37.91 ± 0.11 ***	37.56 ± 0.17 ***	36.98 ± 0.15 ***
No-5	5	35.53 ± 0.11	38.88 ± 0.10 ***	38.33 ± 0.05 ***	37.93 ± 0.07 ***	37.81 ± 0.16 ***
No-5	10	35.33 ± 0.11	38.78 ± 0.19 ***	37.91 ± 0.16 **	37.40 ± 0.17 ***	37.00 ± 0.09 ***
No-11	5	35.38 ± 0.08	38.85 ± 0.16 ***	38.71 ± 0.18	38.68 ± 0.11	38.56 ± 0.16 *
No-11	10	35.28 ± 0.08	38.70 ± 0.11 ***	38.60 ± 0.12	38.53 ± 0.13	38.25 ± 0.09 *
No-14	5	35.40 ± 0.16	38.81 ± 0.12 ***	38.68 ± 0.15	38.61 ± 0.15	38.43 ± 0.13 *
No-14	10	35.93 ± 0.58	38.96 ± 0.12 ***	38.81 ± 0.11	38.56 ± 0.11	38.43 ± 0.12 *
No-13	5	35.53 ± 0.10	38.70 ± 0.16 ***	38.41 ± 0.15	37.96 ± 0.12 **	37.36 ± 0.12 ***
No-13	10	35.56 ± 0.12	38.85 ± 0.11 ***	37.90 ± 0.09 ***	37.28 ± 0.10 ***	37.03 ± 0.09 ***
No-16	5	35.35 ± 0.11	38.75 ± 0.21 ***	38.21 ± 0.07 *	37.90 ± 0.09 **	37.58 ± 0.016 **
No-16	10	35.38 ± 0.18	38.80 ± 0.20 ***	37.83 ± 0.12 **	37.11 ± 0.09 ***	36.83 ± 0.10 ***
No-3	5	35.65 ± 0.11	38.93 ± 0.13 ***	38.93 ± 0.15	38.80 ± 0.13	38.66 ± 0.11 *
No-3	10	35.71 ± 0.11	38.88 ± 0.13 ***	38.73 ± 0.13	38.51 ± 0.07 *	38.60 ± 0.14 *
No-4	5	35.83 ± 0.12	38.90 ± 0.08 ***	38.91 ± 0.12	38.60 ± 0.11	38.48 ± 0.14 *
No-4	10	35.76 ± 0.13	38.91 ± 0.07 ***	38.88 ± 0.14	38.36 ± 0.11 **	38.31 ± 0.11 **
No-1	5	35.66 ± 0.08	38.88 ± 0.13 ***	38.28 ± 0.09 **	37.98 ± 0.15 **	37.43 ± 0.15 ***
No-1	10	35.55 ± 0.16	38.88 ± 0.11 ***	38.13 ± 0.13 **	37.58 ± 0.17 ***	37.06 ± 0.17 ***
No-8	5	35.56 ± 0.23	38.88 ± 0.11 ***	38.80 ± 0.13	38.78 ± 0.13	38.65 ± 0.11 *
No-8	10	35.61 ± 0.18	38.75 ± 0.15 ***	38.53 ± 0.14	38.61 ± 0.11	38.33 ± 0.18 *
No-9	5	35.66 ± 0.12	38.76 ± 0.16 ***	38.75 ± 0.11	38.61 ± 0.13	38.30 ± 0.10 *
No-9	10	35.60 ± 0.12	38.81 ± 0.12 ***	38.76 ± 0.11	38.63 ± 0.13	38.00 ± 0.09 ***
No-10	5	35.83 ± 0.09	38.86 ± 0.14 ***	38.31 ± 0.09 *	37.81 ± 0.13 ***	37.26 ± 0.09 ***
No-10	10	35.78 ± 0.12	38.85 ± 0.15 ***	37.83 ± 0.11 ***	37.40 ± 0.17 ***	37.13 ± 0.18 ***
No-21	5	35.63 ± 0.16	38.85 ± 0.13 ***	38.85 ± 0.12	38.71 ± 0.11	38.53 ± 0.12 *
No-21	10	35.68 ± 0.17	38.86 ± 0.17 ***	38.66 ± 0.12	38.45 ± 0.14	38.20 ± 0.12 *
No-22	5	35.70 ± 0.11	38.81 ± 0.11 ***	38.10 ± 0.10 ***	37.80 ± 0.15 ***	37.20 ± 0.11 ***
No-22	10	35.75 ± 0.09	38.86 ± 0.12 ***	38.23 ± 0.10 **	37.11 ± 0.10 ***	36.73 ± 0.13 ***
**Indomethacin**	4	35.71 ± 0.18	38.95 ± 0.10 ***	37.10 ± 0.09 ***	36.85 ± 0.18 ***	36.33 ± 0.11 ***

All values represent mean ± SEM.* p <0.05, ** p <0.01, *** p <0.001; ANOVA, followed by Dunnett's multiple comparison test. The pre-drug group was compared with the normal group, and the post-drug group was compared with the yeast administration groups.

Table S14. % Inhibition of nitric oxide scavenging activity for the isolated compounds at different concentrations.

Treatments	(% Inhibition ± SD) Concentration				
	**20 μg/ml**	**40 μg/ml**	**60 μg/ml**	**80 μg/ml**	**100 μg/ml**
Compound-2	30.15 ± 14.36	54.43 ± 3.14	63.96 ± 10.70	71.60 ± 10.48	76.83 ± 5.01
Compound-6	10.56 ± 5.34	23.09 ± 3.04	33.50 ± 8.52	50.03 ± 4.00	56.54 ± 6.03
Compound-5	5.16 ± 2.48	10.43 ± 3.25	19.36 ± 8.72	21.63 ± 10.28	27.00 ± 7.85
Compound-11	7.40 ± 4.56	20.06 ± 8.58	36.71 ± 16.53	50.50 ± 10.21	57.00 ± 10.21
Compound-14	17.86 ± 15.85	28.10 ± 22.28	42.40 ± 23.65	59.16 ± 14.84	70.36 ± 14.73
Compound-13	10.40 ± 6.32	14.16 ± 4.35	25.61 ± 5.18	30.04 ± 3.35	33.06 ± 1.86
Compound-16	6.66 ± 2.10	11.26 ± 0.28	19.20 ± 6.40	21.86 ± 5.35	25.61 ± 5.18
Compound-3	7.40 ± 4.56	12.63 ± 5.43	19.43 ± 6.21	28.46 ± 12.00	38.30 ± 5.63
Compound-4	23.86 ± 8.91	41.43 ± 7.77	48.16 ± 7.16	66.03 ± 8.24	77.36 ± 4.22
Compound-1	11.46 ± 5.00	27.90 ± 0.45	44.30 ± 6.76	61.53 ± 2.56	71.66 ± 0.70
Compound-8	7.50 ± 1.55	15.75 ± 0.44	42.61 ± 10.72	50.17 ± 9.22	63.67 ± 12.85
Compound-9	15.06 ± 7.50	29.30 ± 2.98	38.27 ± 2.98	50.43 ± 7.23	63.67 ± 12.85
Compound-10	33.31 ± 19.72	45.38 ± 20.11	56.55 ± 9.42	64.73 ± 4.35	75.26 ± 5.54
Compound-21	39.97 ± 7.66	54.97 ± 6.68	61.70 ± 2.95	63.18 ± 1.66	67.76 ± 5.75
Compound-22	23.86 ± 8.91	41.43 ± 7.77	48.16 ± 7.16	66.03 ± 8.24	77.60 ± 4.22
**Ascorbic Acid**	64.38 ± 15.47	66.64 ± 15.42	78.13 ± 3.22	85.68 ± 1.69	87.23 ± 0.98

The statistical significant was calculated from a one-way ANOVA, and all of the parameters are expressed as mean ± SD of three independent measurements.
